# A Rare Case of Orthostasis, Dizziness, and Non-O1, Non-O139 *Vibrio cholerae* Infection in a Lung Transplant Recipient

**DOI:** 10.1155/2022/9008372

**Published:** 2022-08-26

**Authors:** Michael T. Olson, Tejus Walia, Satish Chandrashekaran, Sreeja Biswas Roy, Ashwini Arjuna

**Affiliations:** ^1^University of Arizona College of Medicine-Phoenix, Phoenix, AZ, USA; ^2^Norton Thoracic Institute, St. Joseph's Hospital and Medical Center, Phoenix, AZ, USA; ^3^McKelvey Lung Transplant Center, Emory University Hospital, Atlanta, Georgia, USA; ^4^Department of Internal Medicine, St. Joseph's Hospital and Medical Center, Phoenix, AZ, USA

## Abstract

Non-O1, non-O139 *Vibrio cholerae* are rare strains that are generally nonpathogenic in immunocompetent hosts. However, this pathogen has been shown to cause gastroenteritis, wound infections, or bacteremia in immunocompromised patients and is associated with significant mortality in these hosts. Herein, we describe a case of hemorrhagic enterocolitis in a lung transplant recipient with an atypical presentation of non-O1, non-O139 *V. cholerae* and ongoing orthostasis. The patient reported recent seafood consumption and was managed appropriately with antibiotic therapy before necessitating esophagogastroduodenoscopy (EGD) for further objective testing.

## 1. Introduction

*Vibrio cholerae* are gram-negative, oxidase-positive, comma-shaped bacilli that primarily inhabit aquatic environments and are classically associated with cholera epidemics [1]. Serogroups of *V. cholerae* are based on the surface O antigen of the lipopolysaccharide. The two major serogroups, O1 and O139, are responsible for epidemic cholera, an acute cause of “rice water” diarrhea. Non-O1, non-O139 *V. cholerae* (NOVC) are genetically distinct strains that can lead to intestinal infections causing gastroenteritis as well as rare extraintestinal manifestations, including wound infections, external otitis, and bacteremia. This noncholeragenic *vibrio* pathogen has been sparsely reported in recent decades but has demonstrated significant morbidity and mortality particularly in the immunocompromised population. The majority of NOVC bacteremia cases have been reported in either cirrhotic or solid-organ transplant patients [2–5]. The clinical presentation in these patients may progress from simple, secretory diarrhea to massive hemorrhagic shock, illustrating the importance of early recognition and prompt treatment in immunocompromised patients. We present a case of hemorrhagic enterocolitis in a lung transplant recipient with ongoing dizziness, orthostasis, and an atypical presentation of NOVC infection. The patient provided informed written consent; this case study was exempt from institutional review board approval.

## 2. Case Presentation

**Figure 1:**
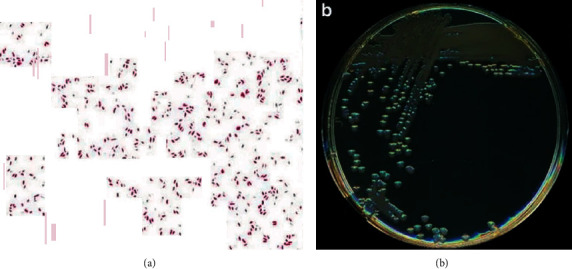
(a) Gram stain (magnification ×1000). (b) Colonial morphology of non-O1, non-O139 *V. cholerae* grown on trypticase soy agar after 18 hours of aerobic incubation at 35°C (reproduced from Deshayes S, Daurel C, Cattoir V et al. Non-O1, non-O139 Vibrio cholerae bacteraemia: case report and literature review. *SpringerPlus*. 2015; 4: 575. Creative Commons Attribution 4.0 International License).

A 47-year-old woman presented to the emergency department at our institution in February 2018 with complaints of severe, watery diarrhea associated with blood, causing dizziness and orthostasis. The patient presented with a previous history of bronchiectasis status/postbilateral sequential lung transplantation nearly four years earlier. Of particular note, the patient reported previous bouts of loose stool in her posttransplant course, which she suspected were caused by mycophenolate mofetil, an agent in her immunosuppression regimen. Because the patient thought that the current diarrheal episode was drug-induced, she did not immediately seek medical attention. She reported a slow symptomatic progression, with increasing frequency of stools now associated with blood. Upon arrival, she was hypotensive with low systolic blood pressure (70-80 mmHg), which responded promptly to intravenous fluids. A gastrointestinal work-up that included *Clostridium difficile* was ordered. The patient was seen by the gastroenterology department and scheduled for an EGD the following morning. Although the patient was started on broad-spectrum antibiotics, the dysentery persisted, prompting admission to the intensive care unit. Six hours later, while being wheeled to the endoscopy suite for EGD at our institution, the laboratory called to report positive *V. cholerae* culture results from a stool sample (Figure 1). The patient's antibiotics were switched to cover this microorganism. After a single dose of doxycycline, the symptoms of orthostasis and dysentery resolved with a dramatic improvement in overall status. On further investigation, the patient and her husband reported recent seafood consumption at a local restaurant the day prior to presenting to the emergency department. The husband was asymptomatic without signs of infection.

## 3. Discussion

*V. cholerae* is a well-defined, gram-negative, oxidase-positive, comma-shaped bacilli endemic to southern Asia and parts of Africa and Latin America [1]. *V. cholerae* is classified on the basis of its surface O antigen of the lipopolysaccharide into serogroups, which are used to distinguish them from the choleragenic species (O1 and O139) responsible for endemic cholera. Infection caused by NOVC strains is not commonly identified in clinical practice, and the mechanism of pathogenicity in these rare strains is even less well-established. In our case, the inability to isolate the strain of this bacteria in subcultures was a limitation, but the dramatic improvement in clinical status after a single treatment dose of doxycycline points toward accurate recognition of NOVC and the need for early intervention in this niche immunocompromised population. Consensus guidelines for the treatment of NOVC infection in the immunocompromised population are still lacking.

In 2015, Chen et al. [6] investigated the clinical and microbiological characteristics of 83 patients with NOVC infections, finding the most common underlying diseases to be liver cirrhosis and diabetes mellitus, followed by malignancy. The most common type of infection was acute gastroenteritis, identified in more than half of patients (54.2%) presenting to the emergency department at two hospitals. In our case report, the patient did not have any abdominal pain or classic symptoms attributable to NOVC infection. It is important for physicians to understand the deleterious effects that a generally nonpathogenic strain of bacteria can have on immunocompromised patients.

In 2009, Patel et al. [4] reported a case of severe sepsis from NOVC in a patient with cirrhosis awaiting orthotopic liver transplant. This case was also identified in a tertiary care center in Phoenix, AZ, USA, located near our institution. An extensive surveillance study performed in the United States over the course of 31 years demonstrated that people are especially at risk for freshwater exposure in Arizona, among other Midwestern and Southeastern states [7]. Therefore, it remains crucial for physicians to warn their immunocompromised patients about the risk of ingesting raw or undercooked seafood in the Southwestern United States, despite popular belief that infection is more common in states with a temperate climate or bordering coastal waters.

## Data Availability

No data were used to support this study.

## Abbreviations

EGD: Esophagogastroduodenoscopy
NOVC: Non-O1, non-O139 *Vibrio cholerae.*
